# The association between farm-level antimicrobial usage and resistance of *Staphylococcus spp.*, as the major genus isolated from aerosol samples, in Japanese piggeries

**DOI:** 10.3389/fvets.2023.1127819

**Published:** 2023-07-24

**Authors:** Sota Kobayashi, Yukino Tamamura-Andoh, Itsuro Yamane, Masahiro Kusumoto, Ken Katsuda

**Affiliations:** ^1^Enteric Pathogen Group, Division of Zoonosis Research, National Institute of Animal Health, NARO, Tsukuba, Ibaraki, Japan; ^2^Division of Hygiene Management Research, National Institute of Animal Health, NARO, Tsukuba, Ibaraki, Japan

**Keywords:** antimicrobial resistance, antimicrobial usage, staphylococci, aerosol, piggery

## Abstract

Bacteria are the dominant particulate matter in livestock houses and can threaten animal and public health. Antimicrobial resistance (AMR) is a crucial concern worldwide, and nationwide measures established based on the One Health approach are being implemented in many countries. This requires multidisciplinary perspectives and collaboration among the human, animal, and environmental sectors. However, information on the AMR risk in livestock house aerosol is limited, especially its association with antimicrobial usage (AMU). Therefore, this study was conducted to reveal the AMR profile of *Staphylococcus*, the major bacterial genus in the aerosol of the piggeries of Japanese farms, and the association between farm-level AMU and AMR. The investigation at 10 farrow-to-finish pig farms revealed that regardless of the sampling season and the piggery group, the resistance rate of isolated staphylococci for oxacillin, erythromycin, and lincomycin was more than 40% of the median and tended to be higher than that for other antimicrobials. The AMU adjusted by the defined daily dose (DDD-adjusted AMU) in the fattening piggery group was significantly higher than that in the sow piggery group (*p* < 0.05). Finally, for the fattening piggery group, the generalized linear mixed model revealed that the AMR rate for oxacillin, erythromycin, tetracycline, and chloramphenicol was positively associated with the corresponding class-based DDD-adjusted AMU of penicillins (odds ratio (OR) = 2.63, *p* = 0.03), macrolides (OR = 6.89, *p* = 0.0001), tetracyclines (OR = 2.48, *p* = 0.04), and amphenicols (OR = 3.22, *p* = 0.03), respectively. These significant positive associations observed in this study imply that the resistance rate for these antimicrobials may decrease by reducing the corresponding antimicrobials’ use. In addition, the resistance rates for erythromycin and chloramphenicol also displayed a positive association with the AMU of antimicrobial classes other than macrolides and amphenicols, respectively. The mechanism underlying these phenomena is unclear; therefore, further evaluation will be needed. As limited studies have reported staphylococci in piggery aerosol and its AMR with quantitative AMU, these results based on on-farm investigations are expected to aid in establishing countermeasures for AMR of aerosol bacteria in pig farms.

## 1. Introduction

Particulate matter in aerosol is an essential indicator of air pollution (1). Toxic and harmful substances, including microorganisms and bacteria, constitute air pollution (2, 3), dominating livestock farm aerosols (4). Therefore, it is rational that bacteria from the environment and animals threaten both animal and public health. For instance, the increased density of animals in piggeries under an intensive production system often results in poor air quality (5). This phenomenon increases the risk of various opportunistic infections, unless ventilation is appropriately managed. Moreover, pig house farmers are at higher risk of respiratory diseases than chicken, cattle, or sheep farmers (6, 7).

Moreover, as the world faces multiple health challenges, antimicrobial resistance (AMR) is a crucial concern listed among the top 10 global health threats (8, 9). A recent worldwide estimation revealed approximately 4.95 million deaths associated with AMR in 2019 (10). Excessive and inappropriate antimicrobial usage (AMU) has been primary reason; therefore, nationwide measures based on the action plan of each country, established based on the Global Action Plan with the One Health approach, are being taken (11). Thus, the human, animal, and environmental sectors need to have multidisciplinary perspectives and collaborate by sharing the insights obtained from each sector.

In Japan, the total quantity of antimicrobials based on the weight of active substances was 1,761.4 tons in 2018. Among those, 36.7 and 12.3% accounted for the livestock sector and feed additives, respectively. Moreover, 74.5% of those for the livestock sector were used in pig production, with tetracyclines, penicillins, sulfonamides, and macrolides as the major classes (12).

Although information on bacterial AMR in the piggery aerosol is available (13), that on its association with AMU is limited. Therefore, this study revealed the AMR characteristics of staphylococci, including animal and human pathogens. We also aimed to evaluate the association between farm-level AMU and AMR of staphylococci. This study’s findings would aid in establishing better countermeasures for AMR in piggeries for animal and public health.

## 2. Materials and methods

### 2.1. Farm recruitment and sampling frame

With the cooperation of the field veterinarians from The Japanese Association of Swine Veterinarians,^1^ consent for participation in this observational study was obtained from ten farrow-to-finish pig farms on a convenient basis. Between November 2017 and July 2020, each farm was visited twice in the warm (spring and summer) and cold (autumn and winter) seasons, respectively, except for farm E (visited only once in the cold season). Brief descriptions of these farms are presented in Table 1 with the varied farm size of 70–1,790, based on sow number. At each visit, aerosol samples were collected from five pig houses of different life stages, including sow stall and farrowing houses as the sow piggery group and the weaners, growers, and finishers houses as the fattening piggery group. Using a commercial air sampler (CORIOLIS MICRO, Bertin Technologies SAS, France) placed at the center of each piggery, 3,000 L of air was passed into 10 mL of sterilized phosphate-buffered saline (PBS, Dulbecco’s PBS (−) “Nissui” Nissui Pharmaceutical Co., Ltd., Tokyo, Japan) for 10 min. These PBS samples were brought to the National Institute of Animal Health for laboratory investigations.

**Table 1:** Brief description of the ten recruited pig farms.

ID	Number of sites	Sows (head)	Annual shipment (head)	Workers	All-in all-out in operation
A	1	70	2,200	3	No
B	2	620	16,200	16	Yes
C	1	1,790	40,700	34	Yes
D	1	510	12,500	11	Yes
E	1	800	18,200	15	Yes
F	2	1,260	21,900	18	Yes
G	2	490	13,000	7	Yes
H	2	320	7,600	7	Yes
I	1	90	1,700	3	No
J	1	240	4,100	6	Yes

**Figure 1:**
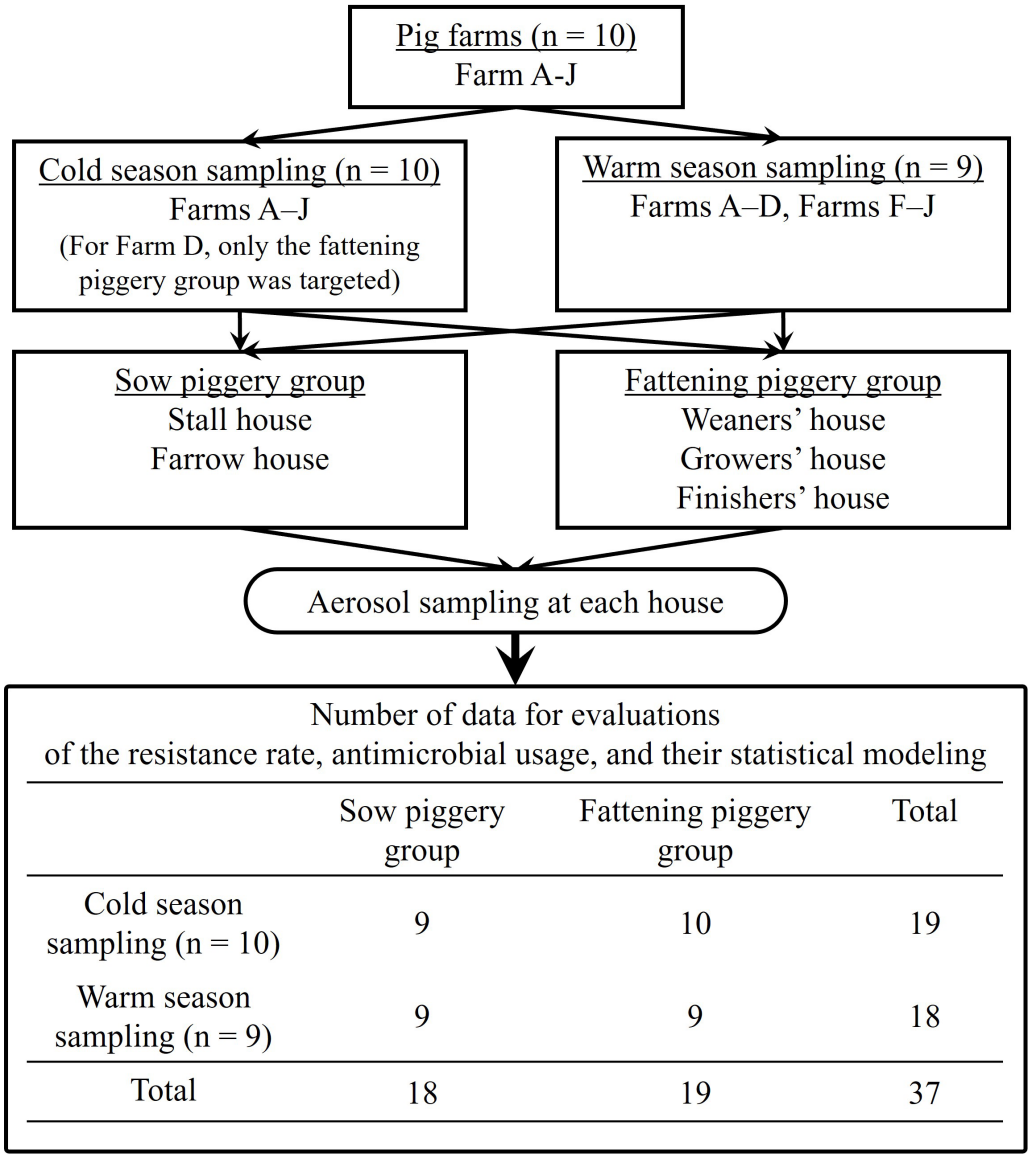
Flowchart of the sampling process in this study based on farm visits by season and piggery group.

In total, 19 sampling visits were made in ten cold and nine warm seasons. Samples were obtained from both piggery groups during all nine warm season visits, meaning nine sow and nine fattening piggeries. However, during the ten cold season visits, due to the technical condition, samples were collected from only the fattening piggery group of Farm D, which meant nine sow and ten fattening piggeries were targeted. This sampling frame is summarized in Figure 1.

### 2.2. Isolation and identification of bacteria

Within 20 h after the on-farm sampling, 100 μL of the PBS sample obtained from each piggery was inoculated on 5% sheep blood in trypticase soy agar (TSA) (BD Trypticase Soy Agar with 5% Sheep Blood, Nippon Becton, Dickinson, and Company, Japan) and mannitol salt agar (MSA) (Mannitol Salt Agar “Nissui,” Nissui Pharmaceutical Co., Ltd., Tokyo, Japan) and aerobically cultured for 16–20 h at 37°C. MSA was used as gram-positive bacteria selective agar, especially, salt-tolerant bacteria, which included some members of the *Staphylococcus* genus. Then, 10 isolates were randomly selected from each medium and stored in 10% glycerol-added Muller Hinton broth (Difco; BD, New Jersey, United States) at −80°C until identification.

All the isolates were identified using species-specific PCR for staphylococci, assumed as the dominant genus by the authors, following previously established procedures (14, 15). For those not identified using this PCR, partial gap gene sequencing (16) or 16S rRNA partial sequencing using a commercial kit (Bacterial 16S rDNA PCR Kit, Takara Bio Inc., Shiga, Japan) was applied following the manufacturer’s instructions. In addition, sequence data were analyzed to determine the most likely species, referring to the EzBioCloud Database.^2^

The identified isolates’ distribution by genus was summarized. In particular, the Chi-square test statistically evaluated the proportion of staphylococci among all the bacterial isolates in each farm by the seasons and the piggery groups.

### 2.3. Antimicrobial susceptibility test

For all the isolated staphylococci, the minimum inhibitory concentration (MIC) values of the 11 antimicrobials below were determined using a commercial kit (Dry Plate “Eiken,” Eiken Chemical Co., Ltd., Japan). Antimicrobial phenotypes were interpreted based on the breakpoints provided by the CLSI guidelines: 0.5 μg/mL for oxacillin (OXA), 0.5 μg/mL for ampicillin (AMP), 8.0 μg/mL for cefazoline (CFZ), 16.0 μg/mL for kanamycin (KAN), 16.0 μg/mL for gentamycin (GEN), 8.0 μg/mL for erythromycin (ERY), 16.0 μg/mL for tetracycline (TET), 32.0 μg/mL for chloramphenicol (CHL), 32.0 μg/mL for vancomycin (VAN), 8.0 μg/mL for lincomycin (LCM), and 4.0 μg/mL for ciprofloxacin (CIP), respectively (17). In addition, the following quality control strains were also assessed: *Staphylococcus aureus* ATCC 29213, *Enterococcus faecalis* ATCC 29212, *Escherichia coli* ATCC 25923, and *Pseudomonas aeruginosa* ATCC 27853.

The resistance rate (%) for each antimicrobial was defined as the proportion of resistant staphylococci isolates among the staphylococci isolates analyzed using the antimicrobial susceptibility test and the distribution of each antimicrobial based on the sampling seasons [*n* = 19 for “cold”s, and 18 for “warm”s (Figure 1)] and piggery groups [*n* = 18 for “sow”s, and 19 for “fattening”s (Figure 1)] was compared using the Mann–Whitney *U* test.

### 2.4. Quantification of AMU

Annual antimicrobial product purchases were recorded for the piggery groups in each farm. This study used the previous year’s volume as the reference AMU data for a farm visit between January and June. The current year’s data were adopted for visits between July and December. Then, the annual mean treatment days (head*day) were estimated as follows:


$$\begin{array}{l} Annualmeantreatmentdaysforsows\;\left(head*day\right)=\\ \;\;\frac{\begin{array}{l} \;\;\;weightofactivesubstanceofeachproduct/\\ \;\;\;\;\;\;\;\;\;\;\;\;\;\;\;defineddailydoseofeachproduct \end{array}}{numberofsows\times \;240kg} \end{array}$$

$$\begin{array}{l} Annualmeantreatmentdaysforfatteningpigs\;\left(head*day\right)=\\ \;\;\;\;\;\;\;\;\;\;\;\;\;\frac{\begin{array}{l} weightofactivesubstanceofeachproduct/\\ \;\;\;\;\;\;\;\;\;\;\;defineddailydoseofeachproduct \end{array}}{numberoffatteningpigs\times 65kg} \end{array}$$

The defined daily dose (DDD) is the Japanese pig production-specific indicator established previously (18, 19), based on the original concept and definition by the World Health Organization (20). These annual mean treatment days were the annual DDD-adjusted usage of each commercial product and summed up by the antimicrobial classes, which were tetracyclines (TETs), amphenicols (APCs), penicillins (PENs), cephalosporins (CEPs), sulfonamides (SULs), pyrimidines (PMDs), macrolides (MCLs), lincosamides (LCMs), aminoglycosides (AGDs), quinolones (QUIs), polymixins (PMXs), and pleuromutilins (PLMs), respectively. The class-based annual DDD-adjusted AMU was statistically compared by the seasons [*n* = 19 of “cold”s, and 18 of “warm”s (Figure 1)] and piggery groups [*n* = 18 of “sow”s, and 19 of “fattening”s Figure 1)], respectively, using the Mann–Whitney *U* test.

### 2.5. Statistical modeling to evaluate the association between the resistance rate of each antimicrobial and class-based annual DDD-adjusted AMU

Association between the resistance rate of staphylococci for each antimicrobial and class-based annual DDD-adjusted AMU was explored using the generalized linear mixed model on each piggery group (*n* = 18 for the sow group and *n* = 19 for the fattening group). Considering the difference in the number of staphylococci successfully obtained on each sampling visit, raw data used for resistance rate calculation were incorporated into the model as the dependent variable; both tested and resistant staphylococci isolates were directly employed. Moreover, with the various farms cooperating, as presented in Table 1, sampling season was forced into the model, and the farm was employed as the random effect. Therefore, the model is described as follows:


$$logit\left(p\right)=log\left(\frac{p}{1-p}\right)=\alpha +season+\sum \beta \chi +RF+e$$

Where p in logit (p) of the model outcome represents the resistance rate accounting for the tested and resistant staphylococci isolates, α is the model intercept, season is the dichotomous data of cold or warm season, χ is the fixed effect as the dichotomous data classified as “high” or “low” based on the median of the class-based annual DDD-adjusted AMU, β is its coefficient, RF is the farm as the random effect, and e is the binomially distributed residual term.

If the resistance rate to an antimicrobial revealed a positive and significant association with “high” class-based annual DDD-adjusted AMU of its class, a multivariable model for the associations with the AMU of other classes was also explored. The final model met the minimum Akaike’s Information Criterion (AIC), and statistical significance was set at *p* < 0.05 for the remaining independent variables with positive fixed effects.

The statistical modeling and other tests mentioned above were performed using R version 4.1.0.^3^ Primarily, the “glmmML” package version 1.1.3^4^ was used for the generalized linear mixed model.

### 2.6. Ethics statement

Animal ethics approval was not required for this study as the samples consisted of piggery aerosol and were collected in the presence of the veterinarians during their routine farm visits for veterinary care and consultation.

## 3. Results

### 3.1. Description of the aerosol bacteria

**Figure 2:**
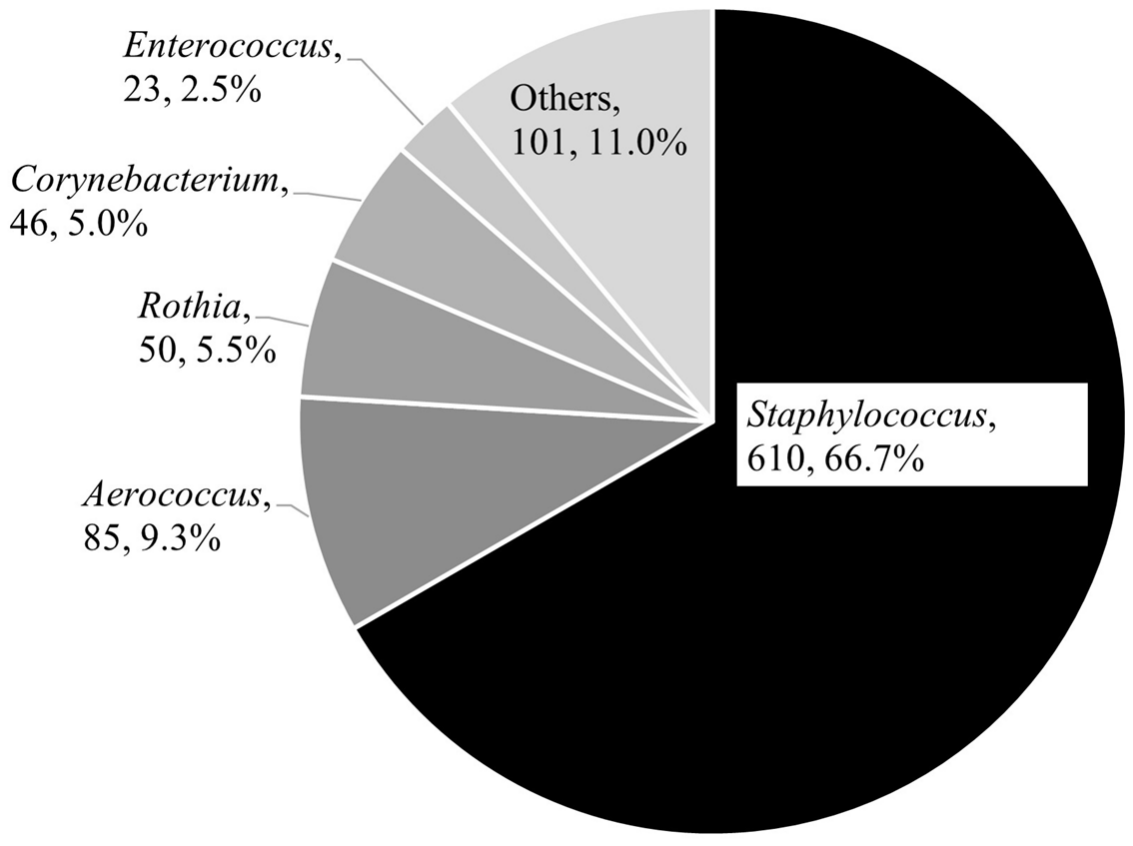
Genus description of bacterial isolates from the aerosol in ten pig farms (*n* = 915 obtained by trypticase soy agar).

In total, 915 bacterial isolates were obtained from TSA, and the genus-level description is summarized in Figure 2. The most dominant genus was *Staphylococcus* (*n* = 610, 66.7%), followed by *Aerococcus* (*n* = 85, 9.3%) and *Rothia* (*n* = 50, 5.5%). Finally, 1,113 staphylococci isolated from TSA (*n* = 610) and MSA (*n* = 503) underwent the antimicrobial susceptibility test, respectively.

**Figure 3:**
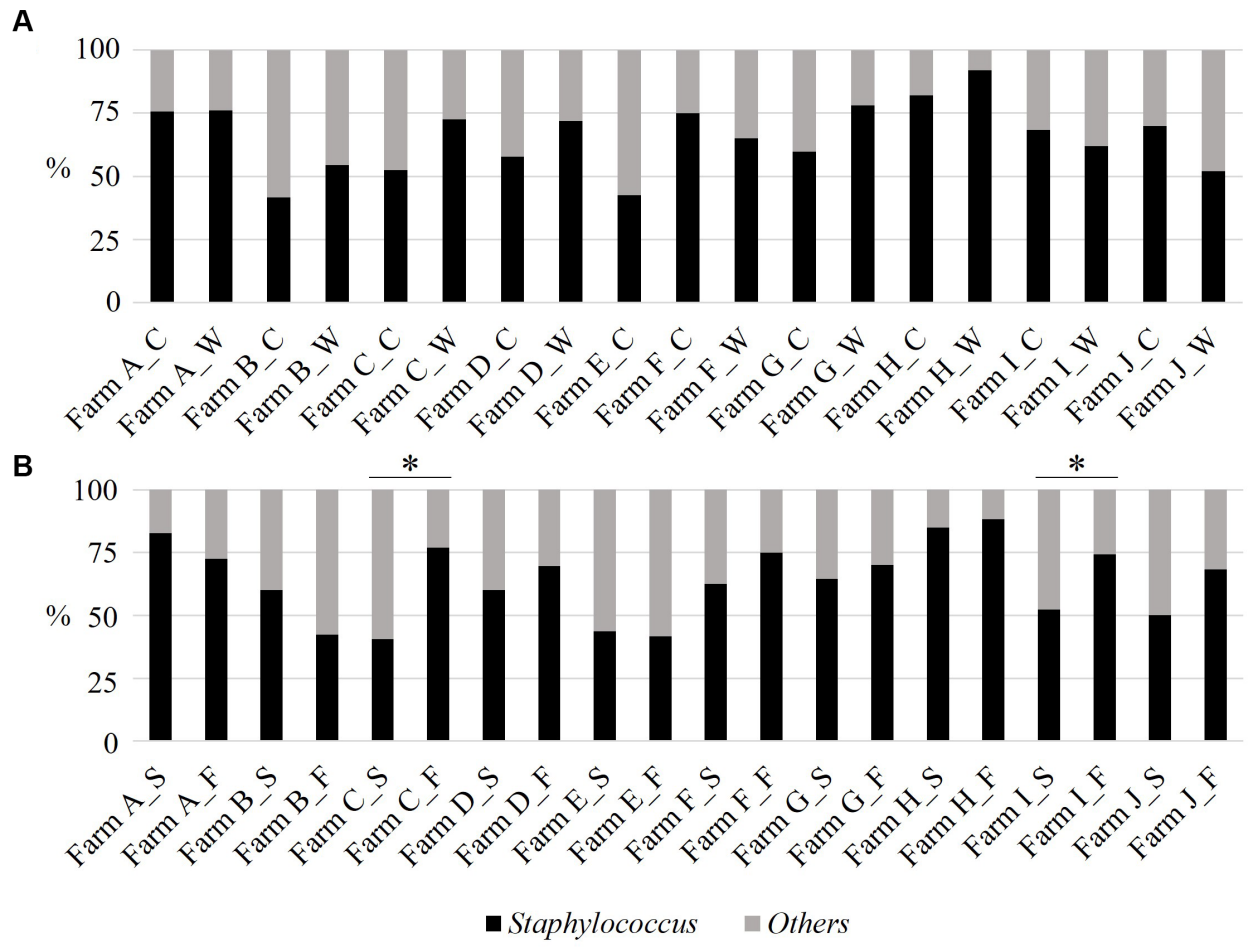
The proportion (%) of staphylococci from the aerosol in ten pig farms by sampling season **(A)** and piggery group **(B)**. The last letter of each item denotes the following: C: cold season, W: warm season, S: sow piggery group, and F: fattening piggery group. *: *p* < 0.05 as revealed by the Mann–Whitney *U* test.

The proportion of staphylococci exceeded 50% in most sampling visits. Farms A and H had over 70%. Apart from Farm E, which was visited once, no intra-farm significant seasonal difference was observed in the staphylococci proportion (Figure 3A, Chi-square test: *p* > 0.05). Sow and fattening piggeries had over 40% of staphylococci among the isolates. Farm A’s sow piggery group and both piggery groups of Farm H had over 80%. Only Farm C and I had significantly higher proportions of staphylococci in the fattening piggery group than in the sow piggery group (Figure 3B, Chi-square test: *p* = 0.02 and 0.03 for Farm C and Farm I, respectively).

Among the 1,113 staphylococci isolates, the most dominant specie was *S. sciuri* (which was renamed *Mammaliicoccus sciuri* in 2020) (*n* = 265, 23.8%), and others had <10% each (Table 2). The top five species *S. sciuri*, *S. cohnii*, *S. saprophyticus*, *S. haemolyticus*, and *S. chromogenes* dominated over 40% of each farm and some over 80% (data not shown).

**Table 2:** Species description of staphylococci isolated from the aerosol in ten pig farms (*n* = 1,113).

Species	Isolates	%
*Staphylococcus sciuri*	265	23.8
*S. cohnii*	98	8.8
*S. saprophyticus*	94	8.4
*S. haemolyticus*	81	7.3
*S. chromogenes*	75	6.7
*S. cohnii subsp. cohnii*	59	5.3
*S. aureus*	52	4.7
*S. simulans*	45	4.0
*S. epidermidis*	36	3.2
*S. hyicus*	35	3.1
*S. nepalensis*	34	3.1
*S. equorum*	31	2.8
Other *Staphylococcus spp.*	208	18.8
Total	1,113	100.0

### 3.2. Distribution of AMR rate

**Figure 4:**
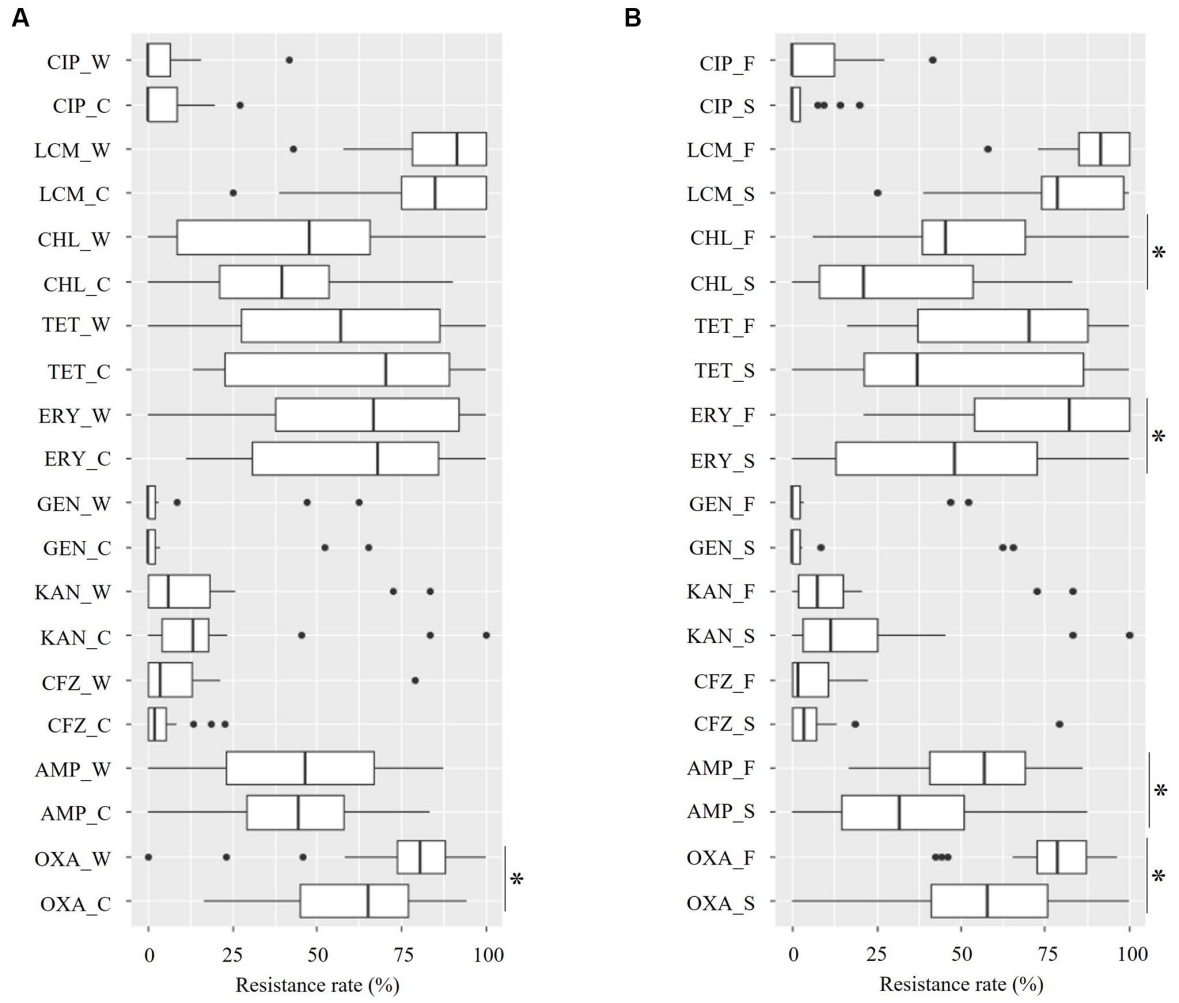
Distribution of the resistance rate for ten antimicrobials of staphylococci from the aerosol in ten pig farms by sampling season **(A)** and piggery group **(B)**. The last letter of each item denotes the following: C: cold season, W: warm season, S: sow piggery group, and F: fattening piggery group. OXA, oxacillin; AMP, ampicillin; CFZ, cefazoline; KAN, kanamycin; GEN, gentamycin; ERY, erythromycin; TET, tetracycline; CHL, chloramphenicol; LCM, lincomycin; CIP, ciprofloxacin. *: *p* < 0.05 as revealed by the Mann–Whitney *U* test.

For 10 of the 11 tested antimicrobials (all the isolates were susceptible to VAN), datasets of the resistance rate of staphylococci by the seasons (Figure 4A and Supplementary file 1) and piggery groups (Figure 4B and Supplementary file 2) were obtained. Regardless of the seasons and piggery groups, resistance rates for OXA, ERY, and LCM were > 40% of the median and tended to be higher than those of other antimicrobials.

A significant seasonal difference was only identified in the resistance rate for OXA, with the median for the cold and warm seasons being 65.4 and 80.7%, respectively (Figure 4A and Supplementary file 1, *p* = 0.03 as revealed by the Mann–Whitney *U* test). In contrast, a significant between-piggery group difference was identified in OXA, AMP, ERY, and CHL. The resistance rates for these four antimicrobials in the fattening piggery group were significantly higher than those in the sow piggery group, with a median of 78.8 and 58.0% for OXA, 57.1 and 31.8% for AMP, 82.4 and 48.4% for ERY, and 45.5 and 21.1% for CHL for the fattening and sow piggery groups, respectively (Figure 4B and Supplementary file 2, all *p* < 0.05 as revealed by the Mann–Whitney *U* test).

### 3.3. Distribution of AMU

**Figure 5:**
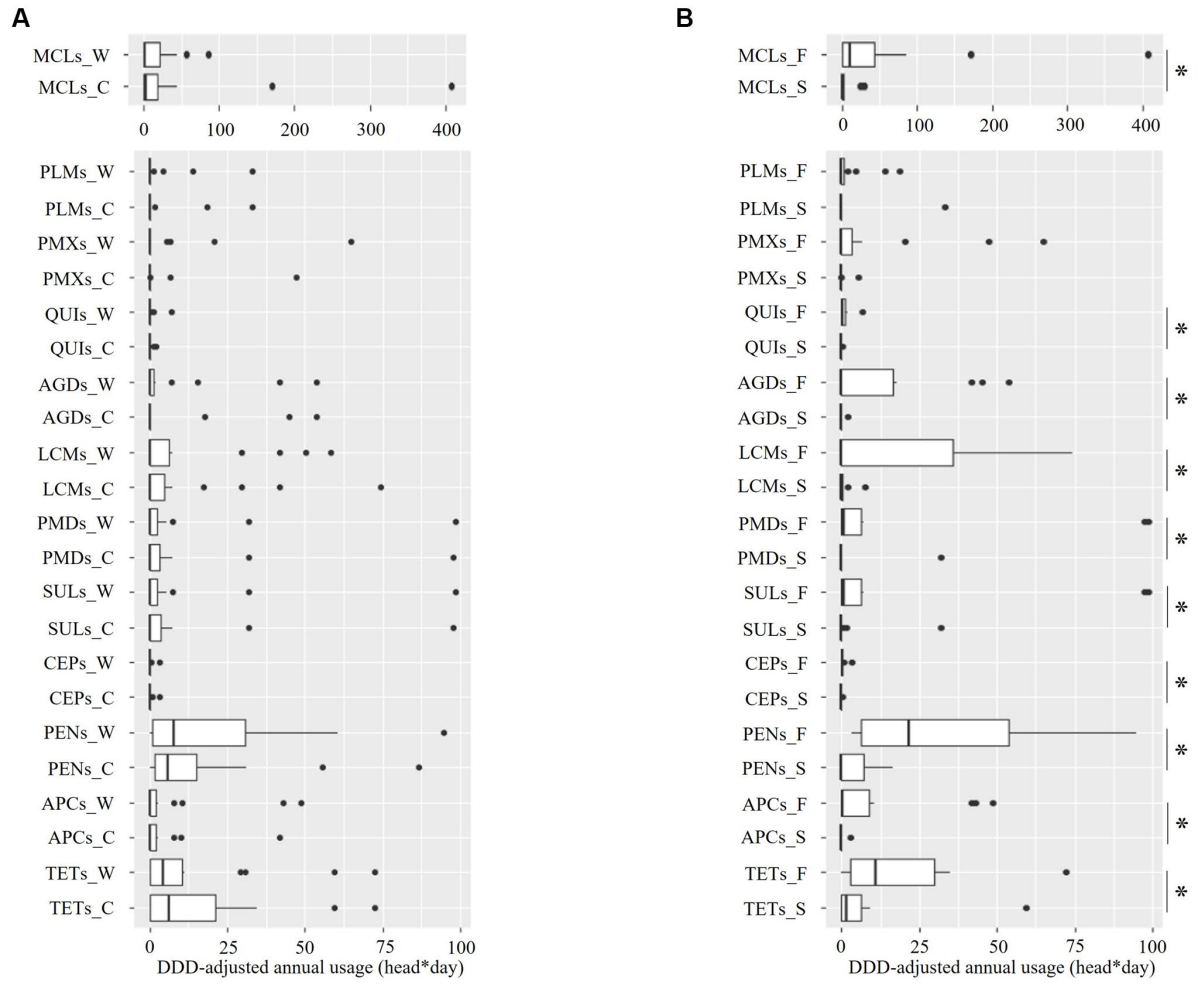
Distribution of the defined-daily-dose-adjusted annual usage of 12 antimicrobial classes in ten pig farms by sampling season **(A)** and piggery group **(B)**. The last letter of each item denotes the following: C: cold season, W: warm season, S: sow piggery group, and F: fattening piggery group. TETs, tetracyclines; APCs, amphenicols; PENs, penicillins; CEPs, cephalosporins; SULs, sulfonamides; PMDs, pyrimidines; MCLs, macrolides; LCMs, lincosamides; AGDs, aminoglycosides; QUIs, quinolones; PMXs, polymyxins; and PLMs, pleuromutilins. *: *p* < 0.05 as revealed by the Mann–Whitney *U* test.

Figure 5 illustrates the distribution of the class-based annual DDD-adjusted AMU. The AMU varied by farm; however, no intra-class difference was identified by season (Figure 5A and Supplementary file 3, all *p* > 0.45 as revealed by the Mann–Whitney *U* test). In contrast, a between-piggery group difference was identified in the AMU of all the classes, except PMXs and PLMs. Therefore, the fattening piggery group had a significantly higher AMU than the sow piggery group, with zero medians for all classes, except TETs and MCLs (Figure 5B and Supplementary file 4, all *p* < 0.05 as revealed by the Mann–Whitney *U* test).

### 3.4. Association between AMU and resistance rate

Table 3 presents four final models obtained by statistical modeling from the datasets of the fattening piggery group for the association between the class-based annual DDD-adjusted AMU and resistance rate of staphylococci. Out of the 11 evaluated antimicrobials, the resistance rate for OXA, ERY, TET, and CHL was significantly associated with the AMU of the corresponding PENs, MCLs, TETs, and APCs, respectively.

**Table 3:** Final models of resistance rate for four antimicrobials of staphylococci from the aerosol in fattening piggeries of ten Japanese pig farms in association with the annual antimicrobial class-based usage.

Model outcome	Significant antimicrobial class	Usage level	Coefficient (SE)	Odds ratio (95% CI)	*p*
Resistance rate of oxacillin	Penicillins	Low	Reference		
		High	0.86 (0.39)	2.36 (1.11, 5.05)	0.03
Resistance rate of erythromycin	Macrolides	Low	Reference		
		High	1.93 (0.51)	6.89 (2.53, 18.73)	0.0001
	Amphenicols	Low	Reference		
		High	1.57 (0.72)	4.81 (1.17, 19.69)	0.03
	Lincosamides	Low	Reference		
		High	3.04 (0.90)	20.91 (3.60, 121.51)	0.001
Resistance rate of tetracycline	Tetracyclines	Low	Reference		
		High	0.91 (0.45)	2.48 (1.03, 5.99)	0.04
Resistance rate of chloramphenicol	Amphenicols	Low	Reference		
		High	1.17 (0.53)	3.22 (1.14, 9.12)	0.03
	Tetracyclines	Low	Reference		
		High	1.22 (0.34)	3.89 (1.73, 6.62)	0.0004

A generalized linear mixed model was used, in which season was forced into each model, and the farm was incorporated as the random effect. SE, standard error; CI, confidence interval.

Regarding OXA, the final model included only PENs, and its “high” usage was associated with a higher resistance rate for OXA [odds ratio (OR) and 95% confidence interval (CI)] = 2.36 (1.11, 5.05), *p* = 0.03). For ERY, the final model included MCLs, APCs, and LCMs. A “high” usage of these three antimicrobial classes was independently associated with a higher resistance rate for ERY (OR (95% CI) = 6.89 (2.53, 18.73), *p* = 0.0001 for MCLs, OR (95% CI) = 4.81 (1.17, 19.69), *p* = 0.03 for APCs, and OR (95% CI) = 20.91 (3.60, 121.51), *p* = 0.001 for LCMs, respectively). For TET, the final model included only TETs, and its “high” usage was associated with a higher resistance rate for TET (OR (95% CI) = 2.48 (1.03, 5.99), *p* = 0.04). In addition, for CHL, the final model included APCs and TETs. A “high” usage of these antimicrobial classes was independently associated with a higher resistance rate for CHL (OR (95% CI) = 3.22 (1.14, 9.12), *p* = 0.03 for APCs, and (OR (95% CI) = 3.39 (1.73, 6.62), *p* = 0.0004 for TETs, respectively). No significant interaction terms were identified in all the final models.

Conversely, analyses of the sow piggery group did not reveal any significantly positive association between the AMU and resistance rate. However, the resistance rates for KAN and TET had a marginally positive association with “high” AGDs and TETs use, respectively (*p* = 0.09 and 0.11, respectively, data not shown).

## 4. Discussion

Previous studies on aerosol bacteria in piggeries have been limited thus far (21); therefore, White et al. (22) evaluated piggery staphylococci for their viability, capturability, inflammogenicity, and biofilm-forming capacity. Eisenlöffel et al. (23) and Tenzin et al. (24) revealed the impact of dust filtration and decontamination. These studies are relevant; however, once countermeasures are in operation, it is better to understand the extent of bacterial distribution and AMR status in these years to strengthen the rationale of the activities. However, few studies have targeted staphylococci AMR with quantitative AMU in Japan. Therefore, this study evaluated the bacterial profile of aerosol in Japanese piggeries, AMR characteristics, and the association between farm-level AMU and AMR, especially for staphylococci.

The aerobic culture using TSA revealed that most isolates were gram-positive bacteria (Figure 2), including the hazardous genus for animal and public health. The most dominant genus was *Staphylococcus*. In this study, staphylococci exceeded 40% and did not differ by sampling season and piggery group in each farm, with a few exceptions (Figure 3). Seasonal differences in sand dust in the general environment influence the bacterial community during aerosol pollution (25); nonetheless, the bacterial distribution stability observed in this study might be due to the relatively steady and closed state in the piggery based on the firm on-farm management system. These results imply the importance of staphylococci among aerosol bacteria and necessitate the maintenance or improvement of on-farm biosecurity levels, especially ventilation and humidity control in piggeries, to prevent clinical diseases in pigs. Further, workers need the shower-in and-out operation and change to washed and clean clothes and disinfected boots before they start their daily tasks. These procedures would promote animal and occupational health.

Among these staphylococci, the most dominant specie *S. sciuri* is a principally animal-associated bacterial species on the skin and mucosal surfaces of various pets and farm and wild animals. However, its clinical relevance in humans is increasing (26), and this bacterium is ubiquitous in human wound infection (27, 28). *S. hyicus* and *S. aureus* are occasionally involved in pig infections (29). Moreover, *S. hyicus* commonly occurs in the nares and on the hairy cutaneous areas of pigs; therefore, it sporadically induces exudative epidermitis in 5–60 d-old pigs along with other staphylococci, such as *S. chromogenes* and *S. aureus* (30). Livestock-associated methicillin-resistant *S. aureus* is more recognized as a public health concern, mainly associated with pigs. In Japan, its presence has been investigated using nasal swabs from slaughtered pigs (31). Given the present situation, there have been few evaluations on the environmental risks of each specie isolated from piggery aerosols. Therefore, a detailed species-based investigation is highly needed under the rational sampling frame in the future.

AMR was revealed for 11 antimicrobials. A high resistance rate of staphylococci was observed for OXA, ERY, and LCM (Figure 3). The influence of season on the resistance rate was not identified in all antimicrobials, except OXA (Figure 4A). The class-based annual DDD-adjusted AMU did not exhibit seasonal differences (Figure 5A). In contrast, the resistance rate in the fattening piggery group was significantly higher than that in the sow piggery group for OXA and AMP of PENs, ERY of MCLs, and CHL of APCs (Figure 4B) as the AMU of the 10 classes, including PENs, MCLs, and APCs, was also higher in the fattening piggery group (Figure 5B). These results indicated that the AMU of the corresponding class might influence some antimicrobials’ resistance compared with environmental conditions. Generally, bacterial survival relies on various factors, such as bacterial species and their burden (32, 33) and environmental conditions, including the type of surface materials, ambient temperature, UV radiation extent, and water and nutrient availability (34, 35). These factors may affect AMR regardless of the bacterial isolates.

From the statistical modeling of the fattening piggery group, the resistance rate for four antimicrobials, including OXA, ERY, TET, and CHL, was positively associated with the AMU of the corresponding class (Table 3). This implies that the resistance rate for these antimicrobials might be decreased by reducing the use of the corresponding antimicrobials.

Moreover, the modeling identified an association between the resistance rate for ERY and the AMU of APCs and LCMs, in addition to MCLs. A similar result was obtained in the association between the resistance rate to CHL and the AMU of TETs, in addition to APCs. The mechanism of these phenomena is unclear; however, Makita et al. (36) suggested that these issues were due to the natural, cross- or co-selection based on analyses of individual pig-originated *Escherichia coli* isolates and qualitative AMU. Further evaluation is strongly needed to validate our study.

In contrast, no significant association between the resistance rate and AMU in the dataset of the sow piggery group was identified. The possible reasons could be the relatively lower AMU in this group, which might be insufficient to establish antimicrobial selection. Moreover, considering that the isolates were from the aerosol, they may include both environmental and pig-origin bacteria. Therefore, the AMR in this group was probably influenced by other factors along with the AMU. However, the resistance rate to KAN and TET displayed a marginally positive association with AGDs and TETs. Among these, TETs with relatively high AMUs in the sow piggery group could be the reason.

Some limitations should be considered in interpreting this study’s results. First, as mentioned above, the AMR of aerosol-origin bacteria is influenced by both the AMU and other factors. Therefore, evaluating the pig-origin (including healthy and diseased ones) staphylococci will help better understand the piggery’s AMR risk. Second, this study’s statistical modeling was performed using aggregated data on the resistance rate and AMU, which could have an ecological fallacy (37). However, antimicrobials are administered on a herd basis in the general pig industry; hence, this is the best way to assess the on-farm situation quantitatively. Based on these results, it is essential to further evaluate the effect of the countermeasures aimed at decreasing the resistance rate for single antimicrobials at the farm level and clarifying multidrug resistance. Lastly, all the evaluations on the association between the resistance rate and AMU were performed on a genus basis to provide an overview of staphylococci. Therefore, detailed investigations focusing on each species will be more useful for the species-level measures.

In conclusion, the aerosol bacteria in Japanese pig farms included those that could threaten public and animal health, mostly staphylococci. Staphylococci resistance to some antimicrobials was associated with using the corresponding antimicrobial class, implying that reducing such antimicrobials would decrease resistance. These results should help establish countermeasures for the AMR of aerosol bacteria in pig farms.

## Data availability statement

The raw data supporting the conclusions of this article will be made available by the authors, without undue reservation.

## Author contributions

KK conceptualized the study. KK and SK designed and performed the on-farm investigation, sample collection, and laboratory experiments. IY, YT-A, and SK contributed to the data management of antimicrobial usage. SK analyzed the data and drafted the manuscript in consultation with KK, YT-A, IY, and MK. All authors contributed to the article and approved the submitted version.

## Funding

This study was conducted under the research project on “Regulatory Research Projects for Food Safety, Animal Health and Plant Protection (grant JPJ008617.17935699/22682153)” funded by the Ministry of Agriculture, Forestry and Fisheries of Japan.

## Conflict of interest

The authors declare that the research was conducted in the absence of any commercial or financial relationships that could be construed as a potential conflict of interest.

## Publisher’s note

All claims expressed in this article are solely those of the authors and do not necessarily represent those of their affiliated organizations, or those of the publisher, the editors and the reviewers. Any product that may be evaluated in this article, or claim that may be made by its manufacturer, is not guaranteed or endorsed by the publisher.

## Footnotes

^1^ http://www.e-jasv.com/

^2^ https://www.ezbiocloud.net/

^3^ https://www.R-project.org/

^4^ https://CRAN.R-project.org/package=glmmML
